# Physical and psychological recovery after vaginal childbirth with and without epidural analgesia: A prospective cohort study

**DOI:** 10.1371/journal.pone.0292393

**Published:** 2023-10-05

**Authors:** Ayumi Maeda, Rimu Suzuki, Rie Maurer, Sumie Kurokawa, Miki Kaneko, Rie Sato, Hiromi Nakajima, Kyoko Ogura, Michiko Yamanaka, Tokujiro Uchida, Yasuko Nagasaka

**Affiliations:** 1 Department of Anesthesiology, Perioperative and Pain Medicine, Brigham and Women’s Hospital, Harvard Medical School, Boston, MA, United States of America; 2 Department of Anesthesia, St. Luke’s International Hospital, Tokyo, Japan; 3 Center for Clinical Investigation, Brigham and Women’s Hospital, Boston, MA, United States of America; 4 Department of Nursing, St. Luke’s International Hospital, Tokyo, Japan; 5 Department of Integrated Women’s Health, Center for Medical Genetics and St. Luke’s International Hospital, Tokyo, Japan; 6 Department of Anesthesiology, Tokyo Medical and Dental University, Tokyo, Japan; 7 Department of Anesthesia, Tokyo Women’s Medical University, Tokyo, Japan; UFPE: Universidade Federal de Pernambuco, BRAZIL

## Abstract

**Background:**

Enhanced recovery is the gold standard in modern perioperative management, including that for cesarean deliveries. However, qualitative and quantitative data on the physical and psychological recovery of women after vaginal childbirth are limited. Whether neuraxial labor analgesia influences postpartum recovery is unknown.

**Methods:**

Primiparous women anticipating a vaginal childbirth between January 2020 and May 2021 were enrolled. Women with major comorbidities or postpartum complications and those who underwent a cesarean delivery were excluded. Daily step count was measured using a wrist-worn activity tracker (Fitbit^TM^ Inspire HR) for 120 hours after vaginal childbirth. Subjective fatigue levels and health-related quality of life were assessed using the Multidimensional Fatigue Inventory (MFI) and EuroQol 5 Dimension 5 Level (EQ-5D-5L), respectively, at the 3^rd^ trimester antenatal visit, on postpartum day 1 and 3, and at the one-month postpartum visit. Rest and dynamic pain scores and the location of pain were documented by participants during postpartum hospitalization.

**Results:**

Among 300 women who were enrolled antenatally, 95 and 116 had a vaginal delivery without (NCB group) and with (EPL group) epidural analgesia, respectively. The median number of steps per 24 hours increased daily in both groups, and no significant difference was detected between the groups. Postpartum pain was mild overall, with median rest and dynamic pain scores being less than 4 and similar between the groups. MFI and EQ-5D-5L scores were the worst on postpartum day 1 in both groups and gradually improved to antepartum level by the one-month postpartum visit. Higher MFI score on postpartum day 1, but not the use of epidural analgesia, was associated with lower odds of achieving adequate postpartum ambulation (defined as >3500 steps between 48 and 72 hours postpartum).

**Conclusion:**

The use of epidural analgesia was not associated with worse recovery outcomes during postpartum hospitalization.

**Trial registration:**

UMIN-CTR, #UMIN000039343, registered on January 31, 2020.

## Introduction

Early mobilization is a key component of enhanced recovery after surgery (ERAS) [1–3]. In addition to its benefit of reducing the risk of thromboembolism and respiratory complications [4,5], ambulation improves physical recovery and pain relief, resulting in shorter postoperative hospital stay and decreased opioid requirements [6]. These benefits have also been demonstrated among postpartum patients who underwent a cesarean delivery [7,8].

Enhanced recovery is especially beneficial among postpartum women who need to take care of a newborn, often breastfeeding while recovering from the physical burden of childbirth. Physical activity during the postpartum period has been shown to reduce the risk of postpartum depression [9] and is recommended by the American College of Obstetricians and Gynecologists [10].

Recently, fitness trackers have been increasingly used for measuring physical activity in healthcare research in various contexts, including during the antepartum and postpartum periods [11–14]. Using a wrist-worn activity tracker, Ma et al. demonstrated that vaginal delivery is associated with greater ambulation than cesarean delivery during the first 24 hours post-delivery [12]. In their study, all participants received neuraxial anesthesia (epidural analgesia for vaginal delivery and spinal anesthesia for cesarean delivery). To date, no data are available on the postpartum ambulatory status of women who underwent an unmedicated childbirth or on postpartum ambulation beyond the first 24-hour period. Although epidural analgesia with high local anesthetic concentration can cause lower extremity motor blockade [15], whether labor analgesia influences postpartum physical recovery is unknown.

The primary objective of our study was to assess physical and psychological recovery and pain burden after a vaginal childbirth using quantitative and qualitative data. We utilized wrist-worn activity trackers to measure participants’ ambulation status and validated questionnaires and self-report diaries to assess their physical recovery, psychological recovery, and acute pain burden during postpartum hospitalization. We also sought to identify the factors associated with better postpartum recovery and assess whether the use of neuraxial labor analgesia affects postpartum recovery outcomes.

## Materials and methods

### Study design

This study was approved by the Institutional Review Board of St Luke’s International Hospital, Tokyo, Japan (IRB# 19-R156) and was registered with the University Hospital Medical Information Network Clinical Trials Registry (UMIN-CTR) registration system (UMIN000039343, PI: Ayumi Maeda, first registered on January 31, 2020). The Strengthening the Reporting of Observational Studies in Epidemiology (STROBE) guidelines for prospective cohort studies were followed.

Healthy primiparous women with singleton, vertex presenting fetuses anticipating a vaginal delivery at St Luke’s International Hospital were enrolled between January 2020 and May 2021. At this hospital with approximately 1400 deliveries (65% vaginal and 35% Cesarean) per year, approximately 70% of women in labor received neuraxial analgesia during the study period.

Participants were approached either during an antenatal or obstetric anesthesia clinic visit after 34 weeks of gestation. Written informed consent was obtained from all participants before enrollment in the study. Those with diseases of pregnancy (e.g., gestational hypertension, preeclampsia, and gestational diabetes), psychiatric disease requiring pharmacologic therapy, chronic pain disorder, contraindications to neuraxial analgesia, hypersensitivity to nickel (used in the wearable device), and known fetal anomalies or complications were excluded. Participants who tested positive for coronavirus disease-2019 (COVID-19) at the time of admission were excluded after initial enrollment. Participants who required intrapartum cesarean delivery were also excluded.

### Data collection

Data were collected during three phases: (1) antepartum visit (after 34 weeks), (2) postpartum hospitalization, and (3) 1-month postpartum visit. Postpartum survey was performed 1 month after delivery because women who had a vaginal childbirth at this hospital had an in-person follow-up visit at 1-month postpartum.

Ambulation data were collected using a wrist-worn activity tracker, Fitbit^TM^ Inspire HR (Fitbit LLC, San Francisco, CA, USA). Approximately two hours after a vaginal delivery, a Fitbit^TM^ Inspire HR was placed on the participant’s wrist on the non-dominant hand by her primary nurse. Ambulation data were collected through the rest of hospitalization, usually until discharge on the fifth day postpartum. The collected data were accessible through the manufacturer’s mobile application and were analyzed using the application programming interface (API).

At the antepartum visit, on postpartum days 1 and 3, and at the 1 month postpartum visit, participants were asked to answer the Japanese versions of two validated questionnaires, the EuroQol 5 Dimension 5 Level (EQ-5D-5L) and Multidimensional Fatigue Inventory (MFI), to assess health-related quality of life and fatigue level, respectively [16–20].

The EQ-5D-5L is a self-reported questionnaire that measures quality of life across five domains: mobility, self-care, usual activities, pain/discomfort, and anxiety/depression. Each dimension is scored on a 5-level severity ranking scale; index values are then calculated using an algorithm. The EQ-5D-5L indices range from -0.224 to 1, where a higher index indicates a better health-related quality of life. EQ-5D-5L also includes a vertical visual analogue scale (EQ VAS) anchored by 0 (the worst imaginable health state) at the bottom and 100 (the best imaginable health state) on the top for respondents to rate their overall health [16–18,21].

The MFI is a 20-item self-reported instrument designed to measure fatigue level. It contains 20 questions with 5-point scales each; thus, total fatigue score ranges from 20 to 100, with a higher score indicating a higher fatigue level [19,20,22].

Antepartum and postpartum pain were evaluated at an antepartum visit (after 34 weeks) and the 1-month post-partum visit, respectively, using a standardized questionnaire created by study investigators. Pain intensity was assessed on the 100 mm Visual Analog Scale (VAS) using anchors of 0 and 100 (no pain and worst pain imaginable, respectively). During postpartum hospitalization, participants recorded rest and dynamic pain scores on a Numeric Rating Scale (NRS) using anchors of 0 and 10 (no pain and worst pain imaginable, respectively) and the location of pain at 2, 6, 12, 18, and 24 hours postpartum, then four times a day subsequently. The options for the location of pain were the perineum, breast, lower abdomen, pelvis, anus, back, head, and others (requires a free comment).

All outcome data were compared between participants who received neuraxial analgesia (EPL group) and those who did not (NCB group). Collected data were anonymized, and the authors did not have access to information that could identify individual participants during or after data collection without referring to the regulatory binder containing a list of participants’ names and medical record numbers with assigned study IDs.

Data on participant demographics, obstetric and neonatal outcomes, oral and intravenous analgesic administration were extracted from electronic medical records.

### Calculation of daily step count

At St. Luke’s International Hospital, most women are discharged home on the fourth or fifth day postpartum after uneventful vaginal delivery. Therefore, we sought to collect ambulation data continuously for 120 hours post-delivery. However, during data analysis it was noted that data from several patients were incomplete (e.g., sporadic data collection or no data recorded). To maintain the quality of data, we included the ambulation data only if the participant wore Fitbit^TM^ for greater than or equal to 20 hours in each 24-hour period, then calculated the adjusted daily step count using the following equation:

Adjusted daily step count = (recorded number of steps)/(duration of data collection in hours) × 24 hours.

If a participant wore Fitbit^TM^ for less than 20 out of 24 hours, the ambulation data were treated as missing data and excluded from the analysis.

### Pain related outcomes

The average of NRS scores recorded by participants over 24 hours was determined, and mean values were used for further analysis. Information on the location of pain was converted to numeric scores based on the frequency reporting over 24 hours. For instance, if a participant reported perineal pain four times, breast pain twice, and lower abdominal pain twice during a certain 24-hour period, scores of 0.5, 0.25, and 0.25 were assigned to each location, respectively (total score of 1 per patient per 24 hours). The scores from all study participants were averaged for each location, and the mean values were used to assess the daily trends in postpartum pain location.

### Intrapartum and postpartum analgesia protocols

For the EPL group, an epidural catheter was placed into the low lumbar interspace, and analgesia was maintained using the programmed intermittent epidural bolus (PIEB) regimen with the following parameters: ropivacaine 1 mg/mL and fentanyl 2 μg/mL, 5 mL intermittent bolus every 60 minutes, 5 mL demand dose, lockout interval of 15 minutes, and 20 mL hourly limit. For breakthrough pain, clinician top-ups (lidocaine 10 mg/mL, mepivacaine 10 mg/mL, fentanyl 50 μg/mL) were administered at the discretion of the primary anesthesia team.

Non-neuraxial analgesia options, such as systemic opioids and inhaled nitrous oxide, were not offered to women in labor in this hospital during the study period. Therefore, women in the NCB group did not receive any analgesics for labor pain relief.

The standard protocol for postpartum pain management included (1) oral acetaminophen (500 mg) as a first-line treatment, (2) oral or rectal diclofenac (25 mg) as a second-line agent, (3) intravenous analgesics (such as acetaminophen and flurbiprofen axetil), and (4) opioids (tramadol and pentazocine). All analgesic agents were administered on a PRN (as needed) basis.

### Statistical analyses

Ma et al. previously demonstrated that step count during the first 24 hours after vaginal delivery with epidural analgesia was 1,205 ± 422 (mean ± SD) [12]. Assuming that a 20% difference in the 24-hour step count would be clinically meaningful [12], we performed a two-sided test of superiority of the 24-hour step count and determined that a sample of at least 31 participants per group will provide 80% power at a 5% significance level (type I error estimate).

Next, we performed an additional two-sided test of superiority of the postpartum pain score at the 5% significance level. Our institutional data had shown that the area under the curve (AUC) of the NRS pain score after vaginal delivery with neuraxial analgesia was 0.21 ± 0.11 (mean ± SD). Assuming that a 20% difference in the NRS-AUC would be clinically meaningful, we determined that a sample size of at least 108 participants per group would provide 80% power at a type I error rate of 5%. To account for drop outs and withdrawals, especially considering the fact that approximately 20% of parturients require cesarean delivery after trial of labor, we enrolled 150 women who were interested in receiving labor epidural analgesia and 150 women who wanted an unmedicated childbirth (300 in total).

Statistical analyses were performed using R statistical software, version 3.5.3 (The R Foundation for Statistical Computing, Vienna, Austria) and EZR (Saitama Medical Center, Jichi Medical University, Saitama, Japan) [23]. Statistical results for categorical data are presented as absolute frequencies with percentages, while results for continuous data are expressed as medians with interquartile ranges (IQR: 25th to 75th percentiles). Chi-square and non-parametric Mann-Whitney U tests were used to evaluate categorical and continuous variables, respectively, between the two groups.

Univariate and multivariate logistic regression analysis was used to identify factors associated with postpartum ambulation outcomes. We defined “adequate postpartum ambulation” as achieving 3,500 steps per 24-hour period between 48 to 72 hours post-delivery, which is typically the time when postpartum women are discharged from hospital after a vaginal delivery in North America. We chose the cut-off value of 3,500 steps per 24-hour period because it is approximately half of the average daily step count of Japanese women of reproductive age [24].

## Results

**Fig 1:**
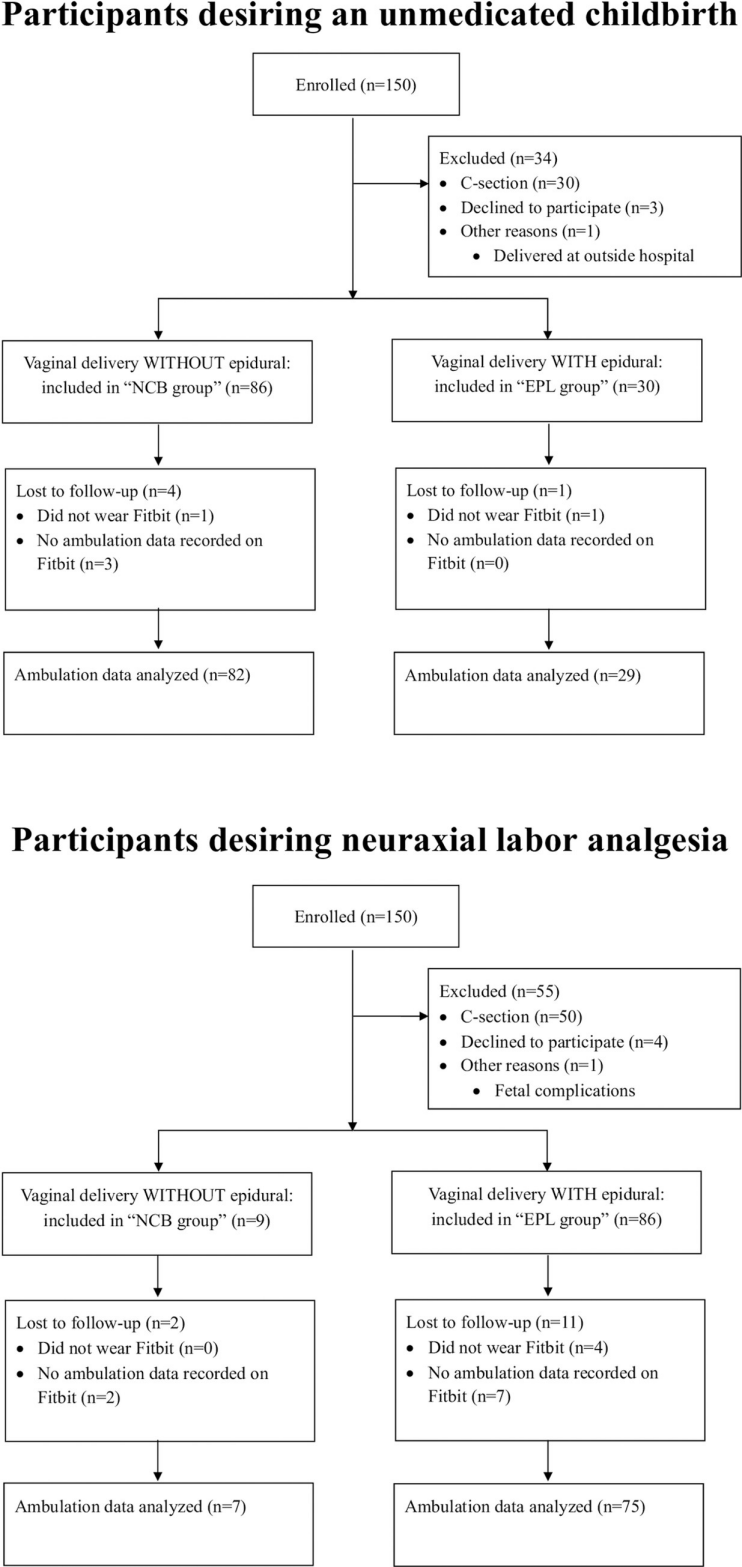
Study recruitment diagram.

A total of 300 women anticipating a vaginal delivery were enrolled in the study, of whom 150 women wanted an unmedicated childbirth and 150 women desired receiving neuraxial labor analgesia. The delivery outcomes of the study participants are shown in Fig 1. Ninety-five women had a vaginal delivery without neuraxial analgesia (NCB group), and 116 women used neuraxial labor analgesia (EPL group).

Baseline characteristics, obstetric outcomes, and neonatal outcomes are shown in Table 1. Participants in the EPL group were older, more likely to undergo induction or augmentation of labor, and had higher incidence of postpartum hemorrhage (blood loss ≥ 500 mL), than those in the NCB group (p<0.05 for all). Differences in the incidences of forceps or vacuum assisted deliveries, episiotomy, or perineal lacerations were not statistically significant. Participants in the NCB group reported better health-related quality of life (assessed by EQ-5D-5L index) and lower fatigue level (assessed by MFI) antenatally.

**Table 1:** Background characteristics and obstetric outcomes.

		NCB (N = 95)	EPL (N = 116)	p value
**Background characteristics**				
	**Age**	**31 [29, 34]**	**33 [30, 36]**	**<0.01**
	BMI at delivery	23.8 [22.3, 25.4]	24 [22.8, 25.7]	0.41
	Psychiatric disorder	1 (1.1)	3 (2.6)	0.76
	Hemorrhoids (pre-existing)	9 (9.5)	11 (9.5)	1.00
**Antepartum questionnaires**				
	**EQ-5D-5L index**	**0.84 [0.77, 0.90]**	**0.82 [0.73, 0.89]**	**0.03**
	EQ VAS	80 [75, 90]	80 [70, 90]	0.12
	**MFI**	**52 [43, 61]**	**60 [46, 67]**	**<0.01**
**Obstetric and neonatal outcomes**				
	Gestational age (weeks)	39.7 [39.0, 40.6]	39.9 [38.8, 40.4]	0.95
	**Induction or augmentation of labor**	**79 (83.2)**	**112 (96.6)**	**<0.01**
	Episiotomy	64 (67.4)	90 (77.6)	0.13
	Vacuum delivery	7 (7.4)	16 (13.8)	0.20
	Forceps delivery	0 (0)	5 (4.3)	0.11
	Perineal laceration	58 (61.1)	80 (69)	0.29
	Perineal laceration degree (0–4)	2 [0, 2]	2 [0, 2]	0.22
	**Blood Loss (estimated or quantitative)**	**444 [342.5, 698.5]**	**570 [425.0, 767.5]**	**<0.01**
	**Postpartum hemorrhage (EBL ≥500 mL)**	**39 (41.1)**	**74 (63.8)**	**<0.01**
	Transfusion	1 (1.1)	1 (0.9)	1.00
	Hemorrhoids (postpartum)	8 (8.4)	13 (11.2)	0.66
	Neonatal birth weight (grams)	2976 [2777, 3176]	3003 [2760, 3333.5]	0.48
	Postpartum length of hospital stay (days)	4.95 [4.78, 5.28]	4.94 [4.81, 5.27]	0.78

Data are shown as n (%), or median [interquartile range].

NCB: Natural Childbirth. EPL: Epidural. BMI: Body Mass Index.

EQ-5D-5D: the EuroQol 5 Dimension 5 Level. MFI: Multidimensional Fatigue Inventory.

VAS: Visual Analogue Scale. EBL: Estimated Blood Loss.

Antepartum, intrapartum, and postpartum pain scores and pain-related outcomes are summarized in Table 2. No difference in pain scores was detected between the groups antenatally, although the EPL group reported slightly higher pain scores at the 1-month postpartum visit.

**Table 2:** Pain related outcomes.

	NCB (N = 95)	EPL (N = 116)	p value
VAS at antepartum enrollment	0 [0, 17.5]	0 [0, 24.5]	0.14
VAS at antepartum phlebotomy	11 [5, 28]	10 [6, 22]	0.60
VAS at postpartum day 1 phlebotomy	7 [3, 17]	8 [4, 19]	0.27
**VAS at 1 month postpartum**	**1 [0, 6]**	**3 [0, 12.5]**	**0.01**
**Highest VAS during childbirth**	**96 [87, 100]**	**86.5 [74, 98.75]**	**<0.01**
Last day of analgesic use (postpartum day)	10 [5, 14]	10 [6, 15.5]	0.33
Still being administered analgesics at 1 month	2 (2.4)	8 (8.2)	0.16

Data are shown as n (%), or median [interquartile range].

NCB: Natural Childbirth. EPL: Epidural. VAS: Visual Analogue Scale.

**Fig 2:**
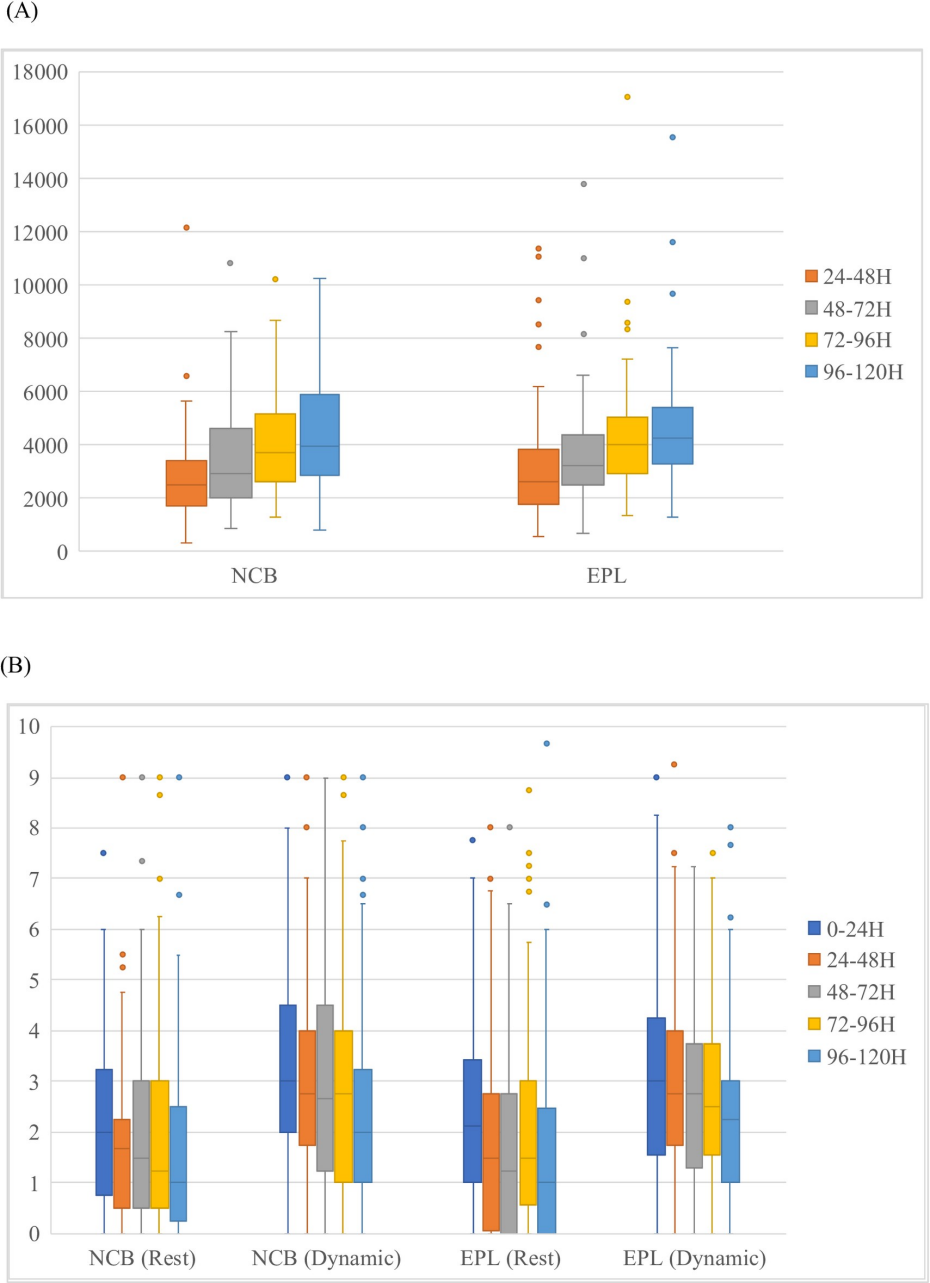
Postpartum trends in (A) adjusted daily steps and (B) Numeric Rating Scale (NRS) pain scores at rest and during movement. Box limits indicate the range of the central 50% of the data, with a central line marking the median value. p > 0.05 for all 24-hour periods in the comparison between the EPL and NCB groups.

Ambulation data from 89 women in the NCB group and 104 women in the EPL group were recorded and included for further analysis (Fig 1). In both groups, the adjusted daily step count increased steadily (Wilcoxon signed-rank test p<0.001 for all day-to-day comparisons), and no statistically significant difference was detected between the groups (Fig 2A). Median pain scores, both at rest and during movement, were less than four in all 24-hour periods, and were not significantly different between the groups (Fig 2B).

Subgroup analysis comparing participants who desired unmedicated childbirth and delivered without labor epidural analgesia (NCB to NCB), those who desired unmedicated childbirth but delivered with labor epidural analgesia (NCB to EPL), those who wanted labor epidural but delivered without labor epidural analgesia (EPL to NCB), and those who wanted labor epidural analgesia and delivered with labor epidural analgesia (EPL to EPL), revealed no statistically significant differences in the adjusted daily step counts and rest and dynamic pain scores in any 24-hour period (S1 Appendix).

Correlation analysis revealed a mild but statistically significant correlation between adjusted daily step count and dynamic NRS between 96 and 120 hours postpartum (Spearman’s rank correlation coefficient -0.22, p = 0.02). This correlation was not detected in other 24-hour periods.

**Fig 3:**
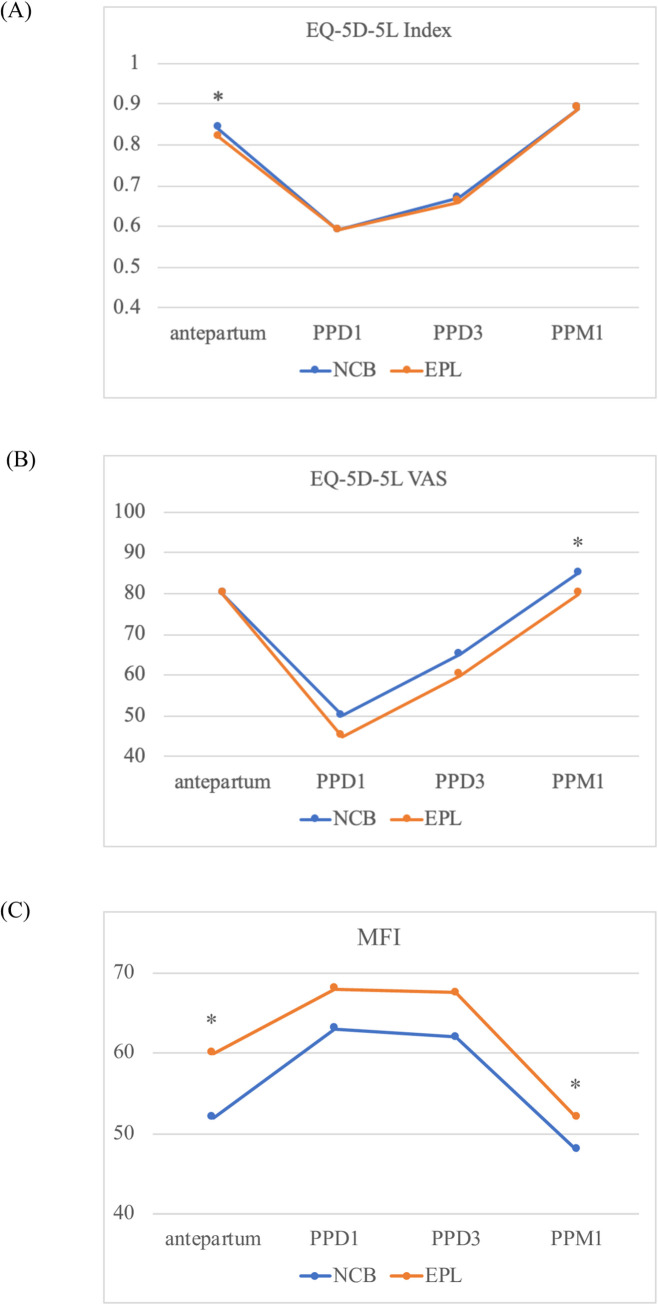
Antepartum to postpartum trends in (A) EQ-5D-5L Index, (B) EQ-5D-5L VAS, and (C) MFI. All data are presented as medians. Asterisks (*) indicate statistically significant differences between two groups (p<0.05). EQ-5D-5L: EuroQol 5 Dimension 5 Level. VAS: Visual Analog Scale. MFI: Multidimensional Fatigue Inventory.

The results of questionnaires on health-related quality of life and fatigue are summarized in Fig 3. In both groups, EQ-5D-5L index and VAS were lowest on postpartum day 1 and gradually improved to antepartum levels by the one-month postpartum visit. Fatigue level assessed by MFI increased during postpartum hospitalization but then improved by one-month postpartum. The EPL group reported lower EQ-5D-5L VAS and higher MFI scores on the one-month postpartum questionnaire than the NCB group.

Among women who had complete ambulation data between 48 and 72 hours postpartum, 33 (39.3%) and 37 (44%) in the NCB and EPL groups, respectively, achieved adequate ambulation, defined as >3500 steps during a 24-hour period. Logistic regression analysis revealed that receiving neuraxial labor analgesia was not associated with increased or decreased odds of achieving adequate ambulation (odds ratio [OR] 1.22, 95% confidence interval [CI] 0.66–2.25). We also built a multivariate model to predict adequate postpartum ambulation. The only factor significantly associated with decreased odds of achieving adequate ambulation was the MFI on postpartum day 1 (adjusted OR 0.97, 95% CI 0.95–0.99).

**Fig 4:**
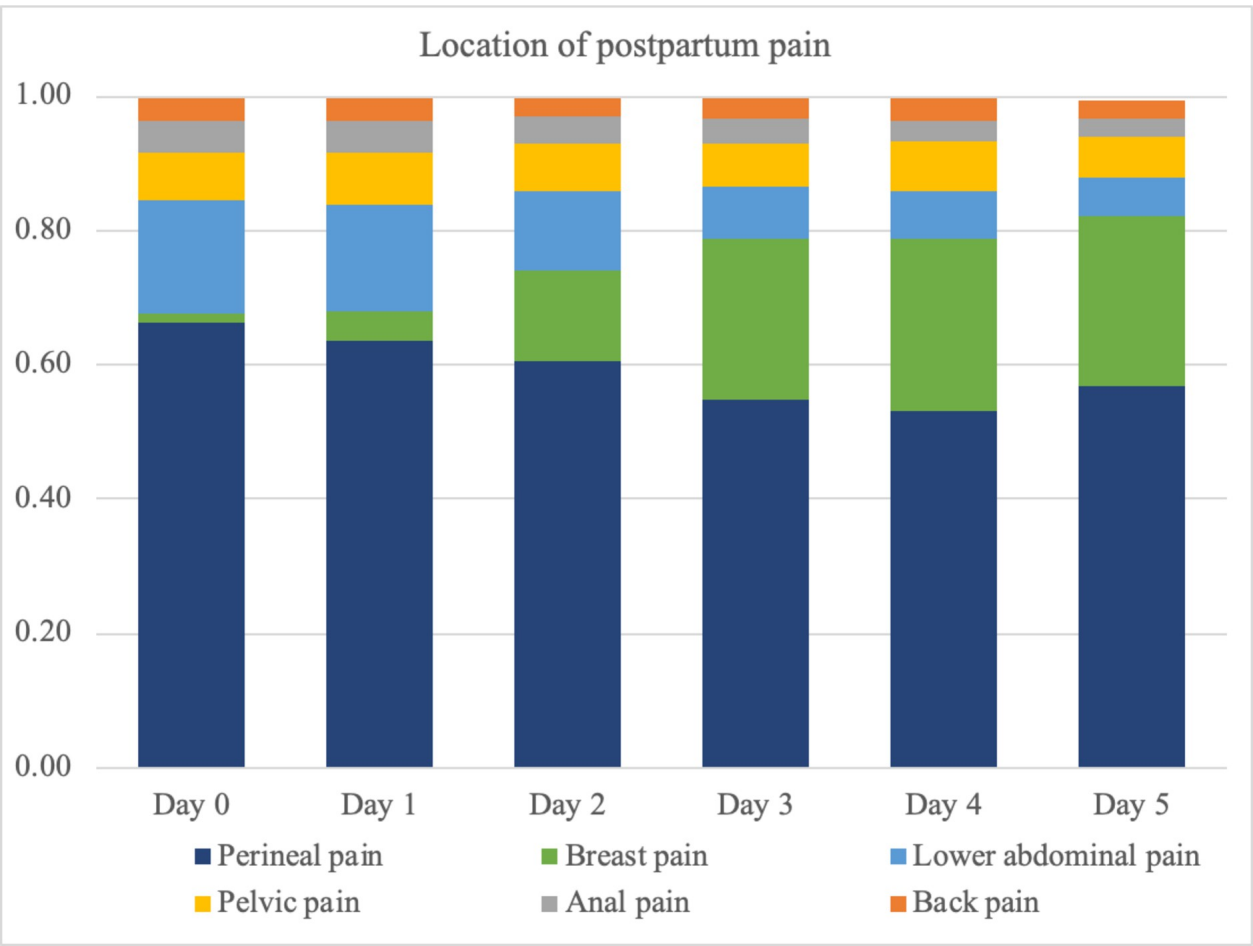
Daily trend in pain location.

Daily trends in the location of postpartum pain are shown in Fig 4. Perineal pain was reported most frequently throughout hospitalization. While lower abdominal pain was also common during the first two days, its frequency trended down beyond postpartum day 2 while breast pain became more prominent (Wilcoxon signed-rank test p < 0.01).

## Discussion

This study aimed to perform a comprehensive assessment of postpartum recovery after vaginal childbirth using qualitative and quantitative data obtained using wrist-worn activity trackers, validated questionnaires (EQ-5D-5L and MFI), and self-reported pain assessment tools. Our study was the first on the trends in daily ambulation, rest and dynamic pain scores, and pain location during postpartum hospitalization to be reported. We found no significant difference in daily step counts between women who used neuraxial labor analgesia and those who did not. Our findings offers additional information for choosing between an unmedicated childbirth and a childbirth using epidural analgesia.

The overall upward trend in daily step count during hospitalization was a reassuring finding. However, only less than half of our participants were able to walk 3,500 steps at 48–72 hours postpartum, which is usually the time when women are discharged from hospital after an uncomplicated vaginal childbirth in North America. Majority of our participants reported lower EQ-5D-5L scores and higher MFI scores on postpartum day 1 and 3 than in the antenatal period. These results suggest that many mothers neither fully recover physically nor psychologically from childbirth before being discharged home; this highlights the need for social support and intervention that allows them to continue to recover at home while caring for their newborns.

While anecdotal evidence has suggested that neuraxial analgesia may help women conserve their energy during labor and delivery, it can cause lower limb weakness which can potentially delay early mobilization [15]. Our study showed that once the effects of neuraxial analgesia have worn off, women who received labor epidural analgesia were able to achieve a level of ambulation similar to that of those who did not, suggesting that labor epidural analgesia is not likely to have a negative impact on postpartum physical recovery.

While the intensity of dynamic pain on the NRS did not decrease dramatically during the 5-day postpartum hospitalization, some changes were noted in the location of pain over time. While the perineum was persistently the primary area of pain, the ratio of women reporting breast pain significantly increased over the five days. This is especially important to recognize in countries where the postpartum length of hospital stay is shorter than that in Japan because breast pain can become a problem after women are discharged from hospital. If the pain becomes a barrier to successful breastfeeding at home, such physical burden can affect the mother’s emotional well-being and lead to postpartum depression [25]. Discharge instructions should include measures to alleviate breast discomfort, safety information on each analgesic option, and when and how to contact a lactation specialist or seek medical care.

Although we initially hypothesized that those with severe pain would have poorer physical recovery [6,26], there was little to no correlation between postpartum pain and daily step count. One potential explanation is that the pain after vaginal delivery is usually milder than postoperative pain after major surgeries; cesarean delivery is associated with greater postpartum pain than vaginal delivery [27,28]. It is possible that pain after vaginal delivery is not severe enough to interfere with postpartum physical activities. Alternatively, early ambulation could have resulted in higher pain scores and offset a negative correlation between pain and ambulation, although no causation can be implied based on our correlation analysis. Sensory and motor blockade caused by neuraxial analgesia could have affected the ability to ambulate in the EPL group. However, this effect should have worn off after 24 hours postpartum; none of the study participants were followed up for postpartum neurologic injury. Postpartum urinary incontinence or retention may influence the level of ambulation. However, studies have shown that neuraxial labor analgesia does not increase the incidence of postpartum urinary incontinence [29,30], and none of our study participants required an indwelling urinary catheter beyond 24 hours postpartum.

We previously published our retrospective study demonstrating that the use of neuraxial labor analgesia was associated with slightly elevated pain scores and analgesic requirement during postpartum hospitalization [31]. This elevation in pain scores in the EPL group was not confirmed in this prospective study. We found a subtle increase in the VAS scores in the EPL group at the 1-month postpartum visit. However, this increase reflects only a two-millimeter difference on a 100 mm scale and is unlikely to be clinically meaningful.

We recognize several limitations inherent in our study. First, ambulation data from several participants were not complete and, therefore, needed to be excluded from the analysis. Using a Fitbit^TM^ at 2 hours postpartum was often difficult when patients were not ready to participate in the study or if there was no available staff who could place the device. This resulted in a limited sample size, especially for the 0–24 hour period and, thus, increased the risk of Type II error.

Participants were encouraged to keep the Fitbit^TM^ device in place throughout their hospitalization, including while showering. However, step count data from some participants were available for only less than 20 hours out of the 24-hour period, likely due to their noncompliance with study instructions and technical faults (i.e., the device not recording the step count). We performed a sensitivity analysis including participants whose data were available for less than 20 hours out of the 24-hour period (S2 Appendix) and found no significant difference between the NCB and EPL groups in any 24-hour period.

Second, our boxplot analysis revealed a few outliers in step counts. One participant in the EPL group (#57) had adjusted daily step counts of 1,748, 13,767, 17,092, and 15,542 in the 24–48 hour, 48–72 hour, 72–96 hour, and 96–120 hour periods, respectively. Her postpartum course was uncomplicated, and it is unknown if her extremely high level of physical activity was intentional. While previous studies have shown a good accuracy of Fitbit^TM^ devices for step counts [11,12,32,33], one systematic review suggested that Fitbit^TM^ devices can overestimate steps in free-living settings [13]. To decrease the risk of overestimation, we conducted a sensitivity analysis to exclude the step count data of this participant. However, no significant difference was detected in the median adjusted daily step counts between the EPL and NCB groups (S3 Appendix).

Third, our sample size calculation was limited by the paucity of literature on postpartum ambulation; the only available literature was on ambulation within the first 24-hour period. Therefore, we expanded our sample size calculation by analyzing data on the NRS outcomes, then enrolled more participants than what was suggested by the sample size calculation to account for patients who required a cesarean delivery. However, it is still possible that our study was underpowered to detect any difference in daily step count between the two groups beyond the first 24 hours.

Comparative analysis between women who used neuraxial labor analgesia and those who had an unmedicated childbirth is inevitably confounded by a variety of factors, especially each woman’s pain perception, when a randomized controlled trial is not performed. The literature has demonstrated that women who utilized neuraxial labor analgesia preferred lower pain intensity at the cost of longer pain duration, while those who had an unmedicated childbirth preferred a shorter labor duration at the cost of higher pain intensity [34,35]. Differences in the background characteristics could have elevated the risk of Type II error in our results.

The Hawthorne effect is another potential bias in our data on postpartum recovery outcomes. All our study participants were fully aware that their step counts were measured. Just by wearing a Fitbit^TM^, they could have been motivated to ambulate more than they would otherwise have. This effect could have biased the findings towards the null.

Lastly, our study participants were asked to wear a Fitbit^TM^ and verbally encouraged to ambulate as tolerated, but we did not set a specific goal for daily step count. Whether encouraging ambulation by setting a specific target will improve physical recovery, decrease postpartum pain, or reduce the risk of postpartum depression requires further investigation.

## Conclusions

We collected a wide variety of data on postpartum physical and psychological recovery using wrist-worn activity trackers, validated questionnaires, and self-reported pain assessment tools. Steady increase in daily step count, improvement in health-related quality of life scores, and decrease in fatigue scores were observed during the 5-day hospitalization after delivery. The use of epidural analgesia was not associated with worse outcomes in physical or psychological recovery after vaginal childbirth.

## Supporting information

S1 Checklist STROBE statement—checklist of items that should be included in reports of observational studies. (DOCX)

S1 Appendix Subgroup analysis comparing participants who desired an unmedicated childbirth and delivered without labor epidural (NCB to NCB), those who desired unmedicated childbirth but delivered with labor epidural (NCB to EPL), those who wanted labor epidural but delivered without labor epidural (EPL to NCB), and those who wanted labor epidural and delivered with labor epidural (EPL to EPL). p<0.05 was considered statistically significant in the analysis using Kruskal-Wallis test. (A) Adjusted daily steps. (B) Numeric Rating Scale (NRS) pain scores at rest and during movement.
(DOCX)

S2 Appendix Postpartum trends in adjusted daily steps using step count data from all participants, including those whose data were available for less than 20 hours out of the 24-hour period. (DOCX)

S3 Appendix Postpartum trends in adjusted daily steps excluding participant #57 in the EPL group. Box limits indicate the range of the central 50% of the data, with a central line marking the median value. p>0.05 for all 24-hour periods for the comparison between the EPL and NCB groups.
(DOCX)

S1 File (PDF)

S2 File (PDF)

## Abbreviations

AUC: Area Under the Curve
CI: Confidence Interval
EPL: Epidural
EQ-5D-5L: EuroQol 5 Dimension 5 Level
ERAS: Enhanced Recovery After Surgery
IQR: Interquartile Range
MFI: Multidimensional Fatigue Inventory
NCB: Natural Childbirth
NRS: Numeric Rating Scale
OR: Odds Ratio
PIEB: Programmed Intermittent Epidural Bolus
UMIN-CTR: University hospital Medical Information Network Clinical Trials Registry
VAS: Visual Analogue Scale
