# Supplementary material for: Physical and psychological recovery after vaginal childbirth with and without epidural analgesia: A prospective cohort study
Source: PLoS One. 2023 Oct 5;18(10):e0292393. doi: 10.1371/journal.pone.0292393 (PMC10553803; doi:10.1371/journal.pone.0292393)
Supplement: S1 Checklist — (DOCX) [file pone.0292393.s001.docx]

STROBE Statement—checklist of items that should be included in reports of observational studies

|  | Item No. | Recommendation | Page  No. | Relevant text from manuscript |
| --- | --- | --- | --- | --- |
| **Title and abstract** | 1 | (*a*) Indicate the study’s design with a commonly used term in the title or the abstract | 1 | Line 1 - 2 |
|  |  | (*b*) Provide in the abstract an informative and balanced summary of what was done and what was found | 3 | Line 31 - 59 |
| Introduction | | | |  |
| Background/rationale | 2 | Explain the scientific background and rationale for the investigation being reported | 5 | Line 66 - 85 |
| Objectives | 3 | State specific objectives, including any prespecified hypotheses | 5 - 6 | Line 86 - 92 |
| Methods | | | |  |
| Study design | 4 | Present key elements of study design early in the paper | 7 | Line 96 - 102 |
| Setting | 5 | Describe the setting, locations, and relevant dates, including periods of recruitment, exposure, follow-up, and data collection | 7 - 8 | Line 103 - 109, 116 - 120 |
| Participants | 6 | (*a*) *Cohort study*—Give the eligibility criteria, and the sources and methods of selection of participants. Describe methods of follow-up  *Case-control study*—Give the eligibility criteria, and the sources and methods of case ascertainment and control selection. Give the rationale for the choice of cases and controls  *Cross-sectional study*—Give the eligibility criteria, and the sources and methods of selection of participants | 7 | Line 103 – 104, 109 – 114 |
|  |  | (*b*) *Cohort study*—For matched studies, give matching criteria and number of exposed and unexposed  *Case-control study*—For matched studies, give matching criteria and the number of controls per case | N/A |  |
| Variables | 7 | Clearly define all outcomes, exposures, predictors, potential confounders, and effect modifiers. Give diagnostic criteria, if applicable | 7 - 10 | Line 116 – 178 |
| Data sources/ measurement | 8* | For each variable of interest, give sources of data and details of methods of assessment (measurement). Describe comparability of assessment methods if there is more than one group | 7 - 10 | Line 116 – 178 |
| Bias | 9 | Describe any efforts to address potential sources of bias | 20, 21 | Line 369 – 372, 389 - 399 |
| Study size | 10 | Explain how the study size was arrived at | 11 | Line 195 - 209 |

Continued on next page

| Quantitative variables | 11 | Explain how quantitative variables were handled in the analyses. If applicable, describe which groupings were chosen and why | 12 | Line 217 – 222 |
| --- | --- | --- | --- | --- |
| Statistical methods | 12 | (*a*) Describe all statistical methods, including those used to control for confounding | 11 – 12 | Line 195 – 222 |
|  |  | (*b*) Describe any methods used to examine subgroups and interactions | 16 | Line 271 - 277 |
|  |  | (*c*) Explain how missing data were addressed | 10, 20 | Line 168 – 169, 366 - 372 |
|  |  | (*d*) *Cohort study*—If applicable, explain how loss to follow-up was addressed  *Case-control study*—If applicable, explain how matching of cases and controls was addressed  *Cross-sectional study*—If applicable, describe analytical methods taking account of sampling strategy | Figure 1 | Loss to follow-up  -> data not analyzed |
|  |  | (*e*) Describe any sensitivity analyses | 20 | Line 369 – 372, 379 – 381 |
| Results | | | | |
| Participants | 13* | (a) Report numbers of individuals at each stage of study—eg numbers potentially eligible, examined for eligibility, confirmed eligible, included in the study, completing follow-up, and analysed | 13, 15,  Figure 1 | Line 224 – 228, 259 – 260,  Figure 1 |
|  |  | (b) Give reasons for non-participation at each stage | Figure 1 | Figure 1 |
|  |  | (c) Consider use of a flow diagram | Figure 1 | Figure 1 |
| Descriptive data | 14* | (a) Give characteristics of study participants (eg demographic, clinical, social) and information on exposures and potential confounders | 13, 14 | Line 232 – 247  Table 1 |
|  |  | (b) Indicate number of participants with missing data for each variable of interest | Fig 1, pg 15 | Figure 1, Line 259 – 260 |
|  |  | (c) *Cohort study*—Summarise follow-up time (eg, average and total amount) | 7 - 10 | Line 116 - 178 |
| Outcome data | 15* | *Cohort study*—Report numbers of outcome events or summary measures over time | 15, 16 | Line 249 – 291, 293 – 295 |
|  |  | *Case-control study—*Report numbers in each exposure category, or summary measures of exposure |  |  |
|  |  | *Cross-sectional study—*Report numbers of outcome events or summary measures |  |  |
| Main results | 16 | (*a*) Give unadjusted estimates and, if applicable, confounder-adjusted estimates and their precision (eg, 95% confidence interval). Make clear which confounders were adjusted for and why they were included | 17 | Line 293 – 300 |
|  |  | (*b*) Report category boundaries when continuous variables were categorized | 12, 16 | Line 218 – 222, 293 - 295 |
|  |  | (*c*) If relevant, consider translating estimates of relative risk into absolute risk for a meaningful time period | N/A |  |

Continued on next page

| Other analyses | 17 | Report other analyses done—eg analyses of subgroups and interactions, and sensitivity analyses | 16, 20 | Line 271 – 277, 369 – 372,  379 – 381 |
| --- | --- | --- | --- | --- |
| Discussion | | | | |
| Key results | 18 | Summarise key results with reference to study objectives | 18 | Line 309 – 316 |
| Limitations | 19 | Discuss limitations of the study, taking into account sources of potential bias or imprecision. Discuss both direction and magnitude of any potential bias | 20, 21 | Line 361 – 403 |
| Interpretation | 20 | Give a cautious overall interpretation of results considering objectives, limitations, multiplicity of analyses, results from similar studies, and other relevant evidence | 18 – 20 | Line 317 – 360 |
| Generalisability | 21 | Discuss the generalisability (external validity) of the study results | 18, 19 | Line 321 – 324, 327 – 330 ,  334 - 336 |
| Other information | |  | | |
| Funding | 22 | Give the source of funding and the role of the funders for the present study and, if applicable, for the original study on which the present article is based | Funding Information | Funding Information |

*Give information separately for cases and controls in case-control studies and, if applicable, for exposed and unexposed groups in cohort and cross-sectional studies.

**Note:** An Explanation and Elaboration article discusses each checklist item and gives methodological background and published examples of transparent reporting. The STROBE checklist is best used in conjunction with this article (freely available on the Web sites of PLoS Medicine at http://www.plosmedicine.org/, Annals of Internal Medicine at http://www.annals.org/, and Epidemiology at http://www.epidem.com/). Information on the STROBE Initiative is available at www.strobe-statement.org.
