# Supplementary material for: Physical and psychological recovery after vaginal childbirth with and without epidural analgesia: A prospective cohort study
Source: PLoS One. 2023 Oct 5;18(10):e0292393. doi: 10.1371/journal.pone.0292393 (PMC10553803; doi:10.1371/journal.pone.0292393)
Supplement: S1 Appendix — p<0.05 was considered statistically significant in the analysis using Kruskal-Wallis test. (A) Adjusted daily steps. (B) Numeric Rating Scale (NRS) pain scores at rest and during movement. (DOCX) [file pone.0292393.s002.docx]

**S1 Appendix**

Subgroup analysis comparing participants who desired an unmedicated childbirth and delivered without labor epidural (NCB to NCB), those who desired an unmedicated childbirth but delivered with labor epidural (NCB to EPL), those who desired labor epidural but delivered without labor epidural (EPL to NCB), and those who desired labor epidural and delivered with labor epidural (EPL to EPL). p<0.05 was considered statistically significant in the analysis using Kruskal-Wallis test.

1. Adjusted daily steps.

| Group | 24 - 48H | 48 - 72H | 72 - 96H | 96 - 120H |
| --- | --- | --- | --- | --- |
| NCB to NCB | 2534 (1697 - 3254) | 2925 (2049 - 4628) | 3688 (2626 - 5097) | 3983 (2908 - 5705) |
| NCB to EPL | 2893 (1748 - 3944) | 3302 (2797 - 4341) | 4202 (3215 - 5861) | 4295 (3609 - 5613) |
| EPL to NCB | 2791 (2340 - 4038) | 2881 (2481 - 4406) | 4074 (2572 - 5351) | 4003 (3062 - 4944) |
| EPL to EPL | 2610 (1779 - 3756) | 3242 (2502 - 4379) | 3996 (2800 - 4679) | 4182 (3283 - 5239) |
| p value | 0.52 | 0.83 | 0.55 | 0.90 |

1. Numeric Rating Scale (NRS) pain scores at rest and during movement.

NRS at rest

| Group | 24 - 48H | 48 - 72H | 72 - 96H | 96 - 120H |
| --- | --- | --- | --- | --- |
| NCB to NCB | 1.8 (0.9 - 2.3) | 1.5 (0.5 - 2.8) | 1.3 (0.7 - 3) | 1 (0.5 - 2.5) |
| NCB to EPL | 1.5 (1.3 - 2.8) | 1.3 (1 - 2) | 1.5 (1 - 2.5) | 1.4 (0.9 - 2.3) |
| EPL to NCB | 2.3 (1.5 - 2.8) | 2 (1.5 - 3) | 1.6 (1.2 - 3.3) | 1.3 (0.6 - 2.5) |
| EPL to EPL | 1.7 (1 - 3.2) | 1.8 (1 - 3) | 2 (1 -3.4) | 1.4 (0.6 - 3) |
| p value | 0.60 | 0.72 | 0.40 | 0.91 |

NRS during movement

| Group | 24 - 48H | 48 - 72H | 72 - 96H | 96 - 120H |
| --- | --- | --- | --- | --- |
| NCB to NCB | 3 (2 - 4) | 2.8 (1.4 - 4.3) | 3 (1.3 - 4) | 2.3 (1 - 3.3) |
| NCB to EPL | 2.8 (2.3 - 3.5) | 2.8 (2 - 3.3) | 2.5 (2 - 3.5) | 2.6 (1.7 - 3) |
| EPL to NCB | 2.5 (2.5 - 3.5) | 2 (1.9 - 4.6) | 2 (1.7 - 4.6) | 1.4 (1.3 - 2.5) |
| EPL to EPL | 3.3 (2 - 4.5) | 3 (2 - 4.4) | 3 (1.9 - 4.1) | 2.3 (1.3 - 3.4) |
| p value | 0.76 | 0.84 | 0.92 | 0.60 |
