# Supplementary material for: Physical and psychological recovery after vaginal childbirth with and without epidural analgesia: A prospective cohort study
Source: PLoS One. 2023 Oct 5;18(10):e0292393. doi: 10.1371/journal.pone.0292393 (PMC10553803; doi:10.1371/journal.pone.0292393)
Supplement: S2 Appendix — (DOCX) [file pone.0292393.s003.docx]

**S2 Appendix**

Postpartum trends in adjusted daily steps using step count data from all participants, including those whose data were available for less than 20 hours out of the 24-hour period.

NCB: Natural Childbirth. EPL: Epidural.

| Group | | 24 - 48H | 48 - 72H | 72 - 96H | 96 - 120H |
| --- | --- | --- | --- | --- | --- |
| Excluding <20 hours | NCB | 2534 (1711 - 3304) | 2923 (2067 - 4605) | 3689 (2626 - 5131) | 3983 (2889 - 5795) |
|  | EPL | 2651 (1773 - 3807) | 3242 (2535 - 4379) | 4026 (2926 - 5055) | 4256 (3310 - 5341) |
| p value | | 0.26 | 0.47 | 0.47 | 0.73 |
| Including <20 hours | NCB | 2568 (1718 - 3524) | 2995 (2084 - 4675) | 3423 (2471 - 5131) | 4270 (2869 - 5886) |
|  | EPL | 2590 (1742 - 3751) | 3173 (2486 - 4383) | 3899 (2748 - 5048) | 4301 (3219 - 5644) |
| p value | | 0.77 | 0.56 | 0.59 | 0.90 |
