# Supplementary material for: Physical and psychological recovery after vaginal childbirth with and without epidural analgesia: A prospective cohort study
Source: PLoS One. 2023 Oct 5;18(10):e0292393. doi: 10.1371/journal.pone.0292393 (PMC10553803; doi:10.1371/journal.pone.0292393)

**S3 Appendix**

Postpartum trends in adjusted daily steps excluding participant #57 in the EPL group. Box limits indicate the range of the central 50% of the data, with a central line marking the median value. p>0.05 for all 24-hour periods for the comparison between the EPL and NCB groups.


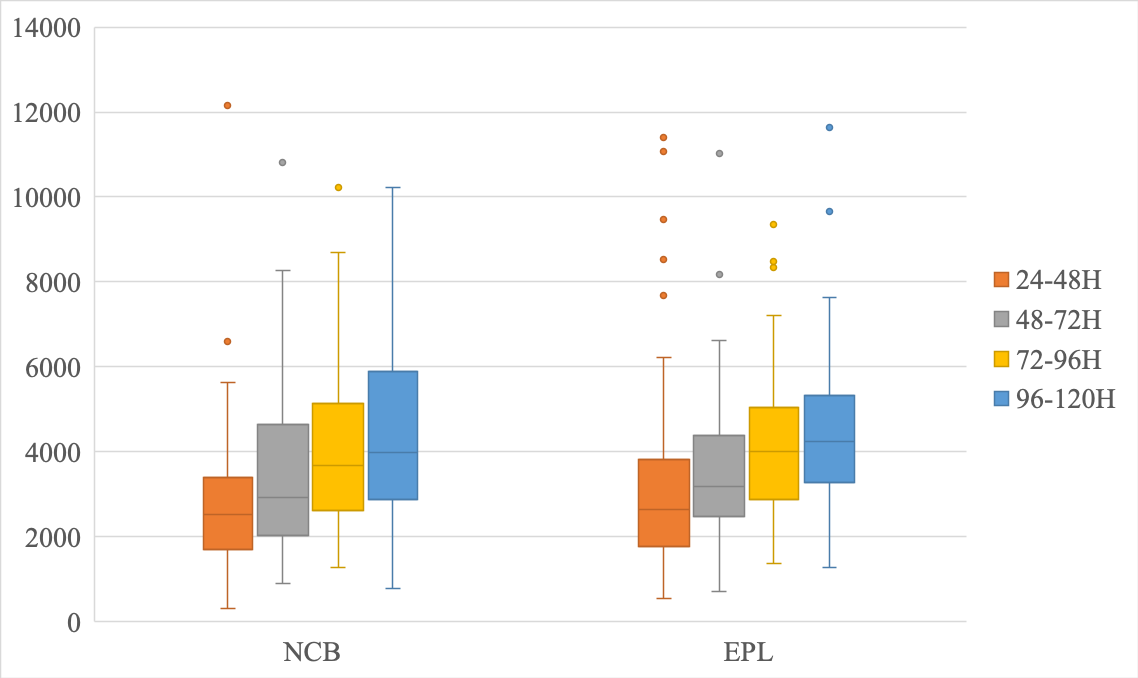

Supplement: S3 Appendix — Box limits indicate the range of the central 50% of the data, with a central line marking the median value. p>0.05 for all 24-hour periods for the comparison between the EPL and NCB groups. (DOCX) [file pone.0292393.s004.docx]
