# Supplementary material for: Physical and psychological recovery after vaginal childbirth with and without epidural analgesia: A prospective cohort study
Source: PLoS One. 2023 Oct 5;18(10):e0292393. doi: 10.1371/journal.pone.0292393 (PMC10553803; doi:10.1371/journal.pone.0292393)
Supplement: S1 File — (PDF) [file pone.0292393.s005.pdf]

St Luke's International University

様式4（所属機関長→申請者）

整理番号	19-R156
区分	□介入研究　　　　■観察研究

西暦2020年01月17日

研究審査結果通知書

研究責任者　　長坂 安子　殿

研究機関の長
聖路加国際大学　学長

依頼のあった研究に関する審査事項について下記のとおり決定しましたので通知いたします。

記

研究課題名	「産褥早期の疼痛や体力回復に対する、産科麻酔の効果に関する定量的検討」（前向き観察研究）
研究責任者	氏名：長坂 安子　　施設名称：聖路加国際病院<br>職種/職位/学年：医師　　所属：麻酔科
審査事項	■研究の実施の適否<br>□研究の継続の適否<br>□重篤な有害事象等<br>□安全性情報等<br>□研究に関する変更<br>□緊急の危険を回避するための研究実施計画書からの逸脱<br>□継続審査<br>□その他（）
審査区分	□委員会審査　（審　査　日：西暦　　年　月　日）<br>■迅速審査　（審査終了日：西暦2020年01月16日）<br>□迅速書類審査　（審査終了日：西暦　　年　月　日）
審査結果	■承認 □修正の上で承認 □条件付き承認 □不承認 □保留 □停止 □中止
「承認」以外の場合の理由等	
コメント	研究対象者が他の研究に参加している方と重複しないよう選定すること。
