# Supplementary material for: Physical and psychological recovery after vaginal childbirth with and without epidural analgesia: A prospective cohort study
Source: PLoS One. 2023 Oct 5;18(10):e0292393. doi: 10.1371/journal.pone.0292393 (PMC10553803; doi:10.1371/journal.pone.0292393)
Supplement: S2 File — (PDF) [file pone.0292393.s006.pdf]

St Luke's International University

様式4（所属機関長→申請者）
Form 4 (From IRB chair to applicant)

整理番号 IRB #	19-R156	
区分Category	☐介入研究 Interventional	■観察研究 Observational

西暦2020年01月17日

研究審査結果通知書
IRB review result notice

研究責任者　　長坂 安子　殿
Dear Yasuko Nagaska

研究機関の長
聖路加国際大学　学長
Dean of St Luke's International University
（印：聖路加国際大学学長印）

依頼のあった研究に関する審査事項について下記のとおり決定しましたので通知いたします。
Below is the decition on the study you applied for the IRB approval.

記

研究課題名 Study Title	「産褥早期の疼痛や体力回復に対する、産科麻酔の効果に関する定量的検討」（前向き観察研究）Physical and psychological recovery after vaginal childbirth with and without epidural analgesia: A prospective cohort study.
研究責任者 Person in charge	氏名：長坂 安子 Name: Yasuko Nagasaka　施設名称：聖路加国際病院 Facility: St Luke's International Hospital<br>職種/職位/学年：医師 Title: MD　所属：麻酔科 Department: Anesthesia
審査事項 Results of the Review	■研究の実施の適否 OK to start this study<br>☐研究の継続の適否<br>☐重篤な有害事象等<br>☐安全性情報等<br>☐研究に関する変更<br>☐緊急の危険を回避するための研究実施計画書からの逸脱<br>☐継続審査<br>☐その他（）
審査区分 Type of Review	☐委員会審査　（審　査　日：西暦　　年　月　日）<br>■迅速審査Rapid Review（審査終了日：西暦2020年01月16日） Review ended in 2020/01/16<br>☐迅速書類審査　（審査終了日：西暦　　年　月　日）
審査結果 Results	■承認 Approved　☐修正の上で承認　☐条件付き承認　☐不承認　☐保留　☐停止　☐中止
「承認」以外の場合の理由等 Reasons for the results other than "Approved"	
コメント Comments	研究対象者が他の研究に参加している方と重複しないよう選定すること。<br>Investigators need to ensure that patients already enrolled in another study will not be enrolled into this study.
